# Mechanism study of the impact of *Escherichia coli* on coal flotation

**DOI:** 10.1371/journal.pone.0272841

**Published:** 2022-08-25

**Authors:** Jianbo Li, Xizhuo Wang, Delong Meng, Ling Xia, Shaoxian Song, Bernardo José Luis Arauz Lara

**Affiliations:** 1 School of Resources and Environmental Engineering, Wuhan University of Technology, Wuhan, China; 2 Instituto de Física, Av. Manuel Nava 6, Zona Universitaria San Luis Potosí, San Luis Potosí, Mexico; 3 Hubei Key Laboratory of Mineral Resources Processing and Environment, Wuhan, China; 4 School of Minerals Processing and Bioengineering, Central South University, Changsha, China; University of Sharjah, UNITED ARAB EMIRATES

## Abstract

*Escherichia coli* as water-borne bacteria exists in the recirculation water of coal flotation and affects the recovery of coal flotation. In order to study the effect of *Escherichia coli* on coal flotation, we changed the concentration of *Escherichia coli* and pH in the coal flotation system and found that *Escherichia coli* had an adverse effect on coal flotation. The concentration of *Escherichia coli* was negatively correlated with the recovery of coal. When the concentration of *Escherichia coli* reached 5.0 × 10^9^ cells/ml, the recovery of coal flotation was 50.25%, and the change of pH basically did not affect the adverse effect of *Escherichia coli* on coal flotation. The mechanism was studied through Zeta potential, Fourier transform infrared spectroscopy, Scanning electron microscopy and Contact angle measurements. The results revealed that *Escherichia coli* could be adsorbed to the coal surface by hydrogen bonding, which changed the hydrophobicity of the coal surface and then reduced the recovery of coal flotation.

## 1. Introduction

Coal accounts for 30% of the global primary energy, and it generates 37% of electricity [1]. In the process of coal utilization, coal preparation can remove inorganic minerals, reduce coal ash and sulfur content. Therefore, coal preparation plays an important role in coal utilization, which can improve coal quality and reduce air pollution [2]. Coal flotation is an important coal preparation technology based on differences of surface properties between hydrophobic low-ash coal particles and hydrophilic high-ash [3]. Coal flotation is the highest water-consuming unit process in coal preparation where water mainly comes from rivers and mines [4]. The river and mine water contains a lot of bacterial spp. The typical bacterial number in river waters can reach 10^6^ to 10^7^ cells/ml [5]. Acidic mine waters were shown to contain higher diversity as microbial numbers reach 106–10^7^ 16S rRNA gene copies/ml [6]. *Escherichia coli* (*E*.*coli*) is a water-borne bacteria. In pure water bodies, the number of *E*.*coli* can reach 10^2^ to 10^3^ cells/ml [7]. But in typical untreated municipal wastewater or groundwater, due to the increase of TDS, Ca and Mg, *E*.*coli* concentration is at a magnitude of 10^5^ cell/ml [8–11]. Since river and mine water is more complex than municipal wastewater or groundwater, higher amounts of *E*.*coil* are expected. Therefore, when river or mine water is used as the water source in the flotation system, a large amount of *E*.*coli* is also introduced. Furthermore, coal flotation with water recirculation can provide favorable conditions for *E*.*coli* growth, given that there is potential nutrition from reagent addition, appropriate oxygen levels and suitable temperature [12]. The accumulation of *E*.*coli* cells in the water recirculation is expected to result in a negative effect on flotation performance [13].

Some studies have explored the role of *E*.*coli* in the mineral flotation process. Wenying Liu studied the influence of *E*.*coli* on the flotation of chalcopyrite and found that the attachment of *E*.*coli* to the surface of chalcopyrite affected the attachment of the collector to the particles, resulting in a decrease in the hydrophobicity of the mineral surface, and ultimately reducing the recovery rate and flotation efficiency [14]. At the same time, Wenying Liu found that disrupted *E*.*coli* had a negative effect on copper as well as gold flotation [15]. Tao wu and Mohsen Farahat studied the adsorption of *E*.*coli* onto quartz and found that *E*.*coli* confer hydrophobic properties to quartz and the biotreated quartz was positively charged, so a large amount of the collector was adsorbed and the recovery increased [16,17]. Due to the different effects of *E*.*coli* on minerals, *E*.*coli* can act as a collector for specific minerals, separating the target mineral from impurities. Mohsen Farahat’s research found that using *E*.*coli* could separate quartz from a hematite–quartz mixture [18].

Compared with the study of *E*.*coli* on the flotation of minerals such as quartz and chalcopyrite, there are relatively few studies on coal flotation. In coal bioflotation, a number of studies focused on the use of bacteria as surface modifiers on coal flotation such as *Paenibacillus polymyxa*, *Rhodotorula mucilaginosa*, etc [19]. However, far less is known about the impact of water-borne bacteria on coal flotation. To understand this, we investigated the effect of *E*.*coli* concentration and pH on the recovery of coal flotation. The adsorption of *E*.*coli* on the coal surface was confirmed by Scanning electron microscopy (SEM) and adsorption mechanism was studied by Contact angle measurements, Zeta potential and Fourier transform infrared spectroscopy (FTIR).

## 2. Experimental

### 2.1. Coal sample

The coal sample was obtained from Taixi, China. The coal sample was crushed and wet ground with a ball mill. The -75 + 38 μm fraction was used for floatation tests, SEM, Contact angle and FTIR measurements. The -10 μm fraction was used for Zeta potential tests.

### 2.2. The bacteria strain

A pure culture of *E*.*coli* strain was cultured in Luria-Bertani (LB) medium (tryptone (10 g/l), yeast extract (5 g/l), NaCl (10 g/l)) and grown in flasks for 16 h at 130 rpm, 37°C. Then the cells were harvested by Heraeus multifuge X1R centrifugation (Thermo Scientific) at 5000 r for 5 min. Finally the cell pellets were suspended in the sterilized distilled water after being washed three times. The bacterial cell concentration was measured by optical density (OD_600_) using an Orion Aquamate 8000 (Thermo Scientific), where 1 OD equals 1 × 10^9^ cell/ml.

### 2.3. Flotation experiments

The RK/FGC35 laboratory flotation machine (Rock Machine Factory, Wuhan, China) was used in flotation tests. The flotation cell was 0.1 L. In each experiment, 4 g of coal particles and a known concentration of *E*.*coli* were conditioned in 80 ml of distilled water at a specific pH value for 15 min. The desired pH value was regulated by HCl (0.1 mol/l) and NaOH (0.1 mol/l). Then the frother Methyl Isobutyl Carbinol (MIBC) (200 g/t) was added to the flotation cell and stirred for 3 min at 1600 r/min. The flotation concentrates were filtered, dried at 60°C in a dryer and weighed for analysis.

### 2.4. Zeta potential measurements

Malvern Zetasizer Zeta-Nano (Malvern, UK) was used to test the Zeta potential of the coal and *E*.*coli* at the same ionic strength (10^3^ M KNO_3_). Zeta potential was measured by the combination of electrophoresis and laser Doppler velocimetry. In order to measure zeta potential of coal after interaction with *E*.*coli*, the sample was first conditioned with *E*.*coli* under the required conditions (pH, adsorption time and cell concentration).

### 2.5. FTIR measurements

Fourier transform infrared spectroscopy (FTIR) measurements of the coal and *E*.*coli* was conducted using a Nicolet FTIR-6700 spectrometer (Thermo USA). All measurements were conducted at room temperature (25 ± 1°C) and the background spectrum was obtained using KBr pellets. The coal sample was conditioned with *E*.*coli* under the required conditions (pH, adsorption time and cell concentration), dried in the air and used for measurement.

### 2.6. Scanning electron microscopy

SEM studies were carried out to observe *E*.*coli* attachment to the coal surface. After adsorption under suitable conditions (pH, adsorption time and cell concentration), air drying and coating with gold under vacuum using an ion coater, images were then acquired using a Phenom Prox (Phenom Scientific, Netherlands) scanning electron microscope.

### 2.7 Contact angle measurements

The contact angle measurements were carried out through a Contact angle detector (JC2000C1) using the captive bubble method. The coal was taken before and after interaction with the *E*.*coli* suspension at the required conditions (pH, adsorption time and cell concentration), dried in the air. Then the contact angle measurements were carried out.

## 3. Results and discussion

### 3.1 Flotation

**Fig 1:**
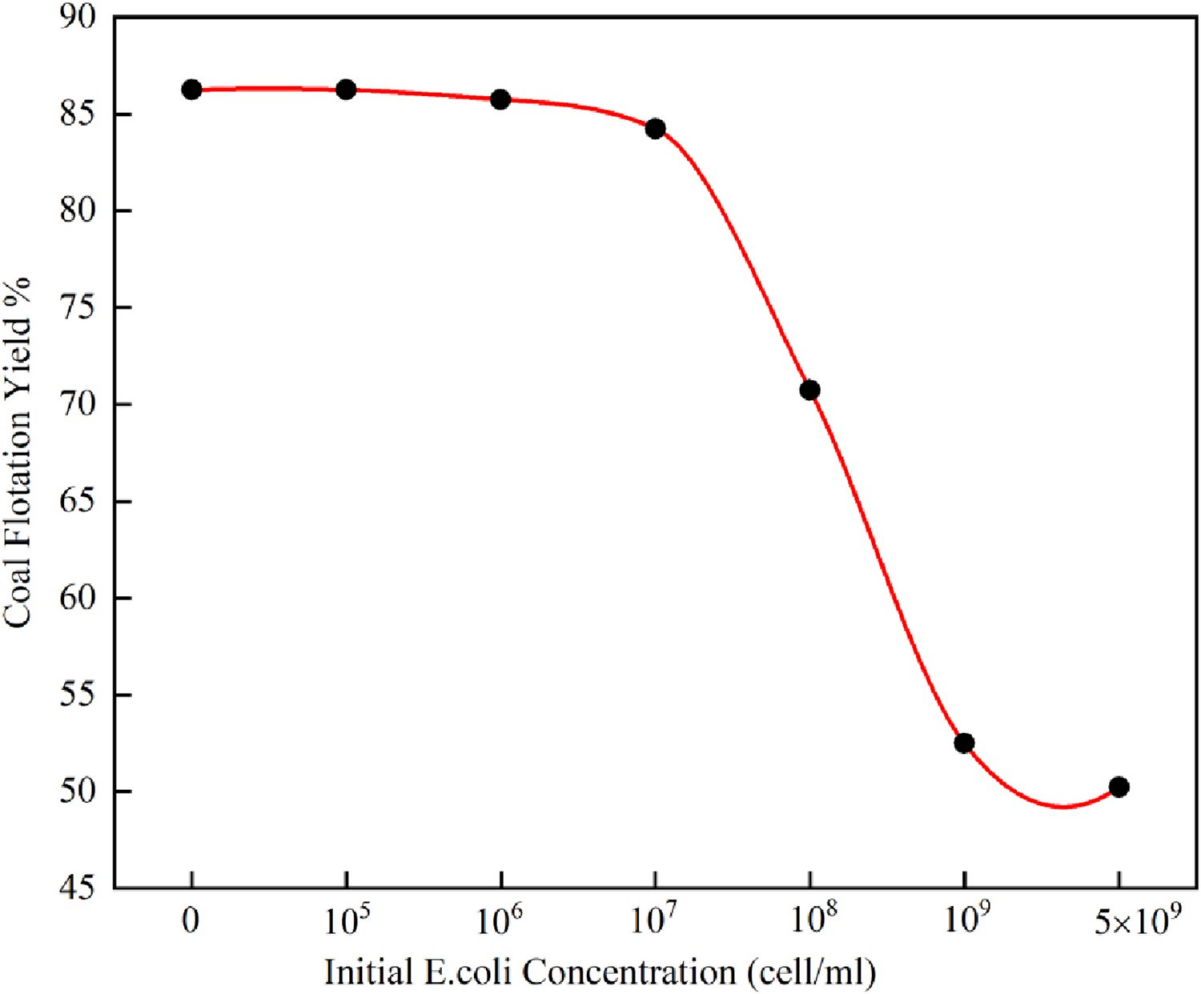
Effect of *E*.*coli* concentration on flotation recovery of coal.

Fig 1 shows the effect of *E*.*coli* concentration on the flotation recovery of coal. The experiments were carried out at pH 6 in the presence of distilled water for a conditioning time of 15 min and a flotation time of 3 min. Due to the great hydrophobicity and high flotation recovery of coal, and in order to further study the effect of *E*.*coli* on coal surface without considering the effect between *E*.*coli* and the reagent, no collector was added in this test. In Fig 1, the flotation recovery of coal decreased as the concentration of *E*. *coil* increased. The recovery of coal could reach 86.25% in the absence of *E*. *coli* from the flotation process, but only 50.25% of the coal could be recovered when 5 × 10^9^ cells/ml existed. The increase in cell concentration decreased the flotation recovery of coal. This result was similar to the effect of *E*.*coli* on the flotation of other minerals. Wenying Liu studied the quantitative relationship between the *E*.*coli* concentration and the flotation performance of chalcopyrite and pyrite, and found that chalcopyrite and pyrite recovery decreased with increasing bacterial concentration [12].

**Fig 2:**
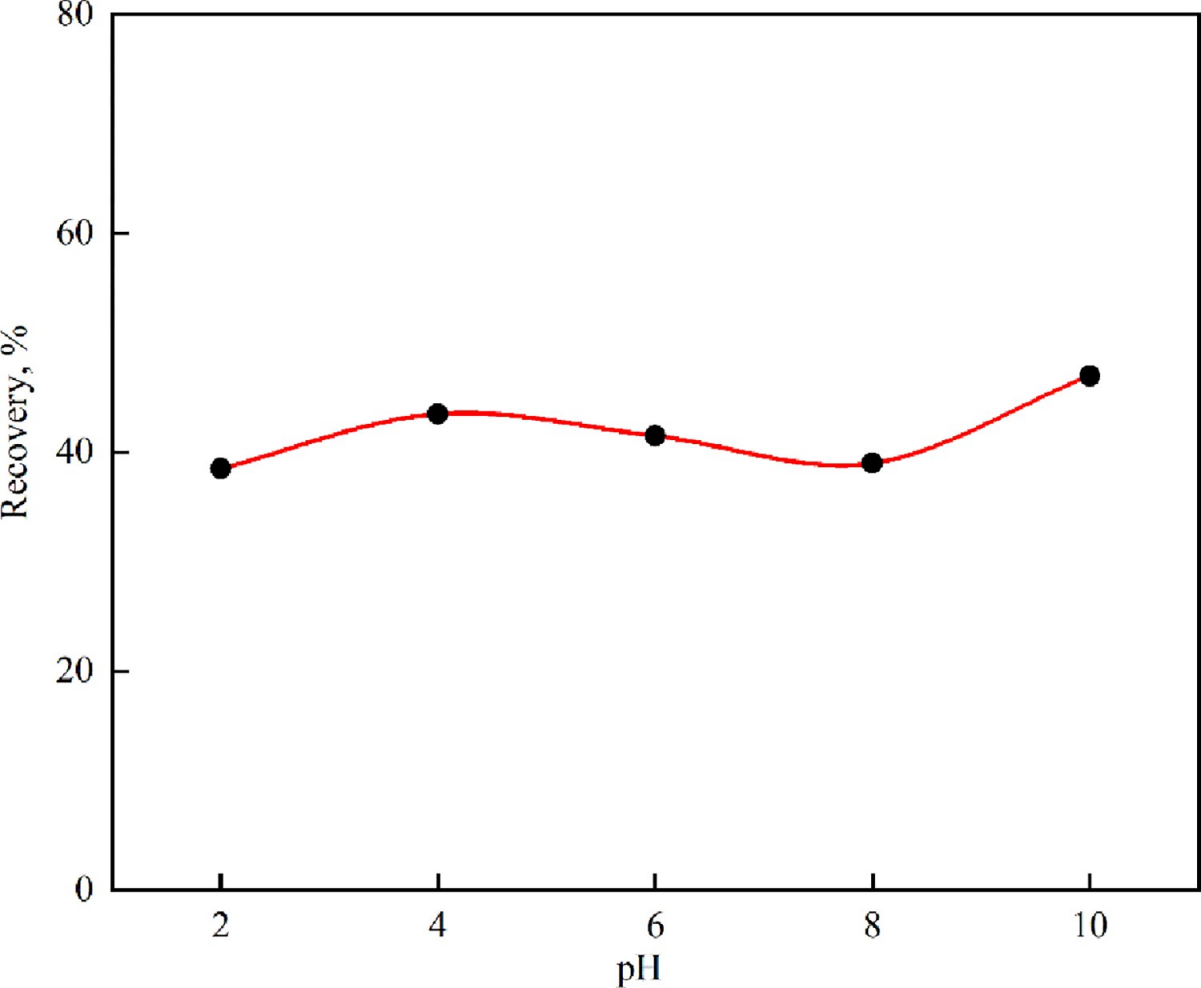
Effect of pH on flotation recovery of coal with *E*.*coli* at concentration of 5 × 10^9^ cells/ml.

In order to explore the effect of pH on coal bioflotation, bioflotation tests at different pH values were carried out, and the results are shown in Fig 2. Experiments were carried out at an *E*.*coli* concentration of 5 × 10^9^ cells/ml in the presence of distilled water for a conditioning time of 15 min at different pH values and a flotation time of 3 min. The results showed that the pH value had little effect on the bioflotation of coal. When the pH = 2–8, the bioflotation recovery of coal was about 40%, when pH = 10, the bioflotation recovery of coal was 47%. Therefore, compared with pH = 2–8, the adverse effect of *E*.*coli* on coal flotation was relatively reduced when pH was 10, which may be due to the fact that the high pH is not conducive to the growth of *E*.*coli*.

### 3.2 Scanning electron microscopy

**Fig 3:**
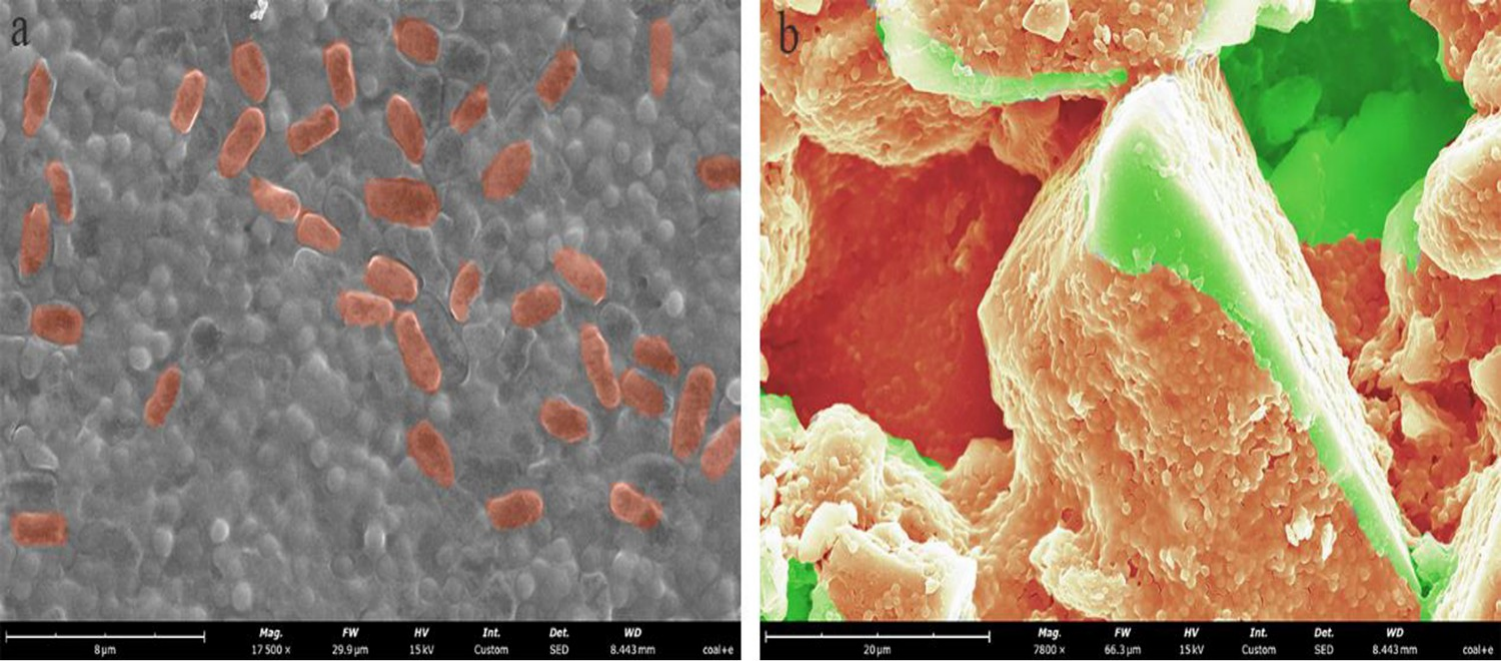
Scanning electron micrograph showing (a) *E*.*coli* cells and (b) coal interaction with *E*.*coli*.

The adsorption of *E*.*coli* on coal surface can be observed by SEM. Fig 3(A) shows a SEM image of *E*.*coli* cells. It revealed that *E*.*coli* is a rod-shaped bacteria of 3μm length. Fig 3(B) shows a SEM image of coal after biotreatment with 5 × 10^9^ cells/ml of *E*.*coli* at pH 6. In Fig 3(B), the orange and green parts show the areas where coal surface is adsorbed and not adsorbed with *E*.*coli* respectively. It could be seen from Fig 3(B) that the coal surface was basically covered by *E*.*coli*. Generally, the bacterial adsorption is based on the electrical potential and hydrophobicity [20]. And the adsorption of bacteria starts with transportation, followed by contact and initial adhesion, firm attachment and surface colonization [21]. When bacteria was adsorbed to the mineral surface, the electrokinetic and hydrophobicity properties of the mineral surface were subsequently changed, and that affected the flotation effect [22].

### 3.3 Contact angle

**Fig 4:**
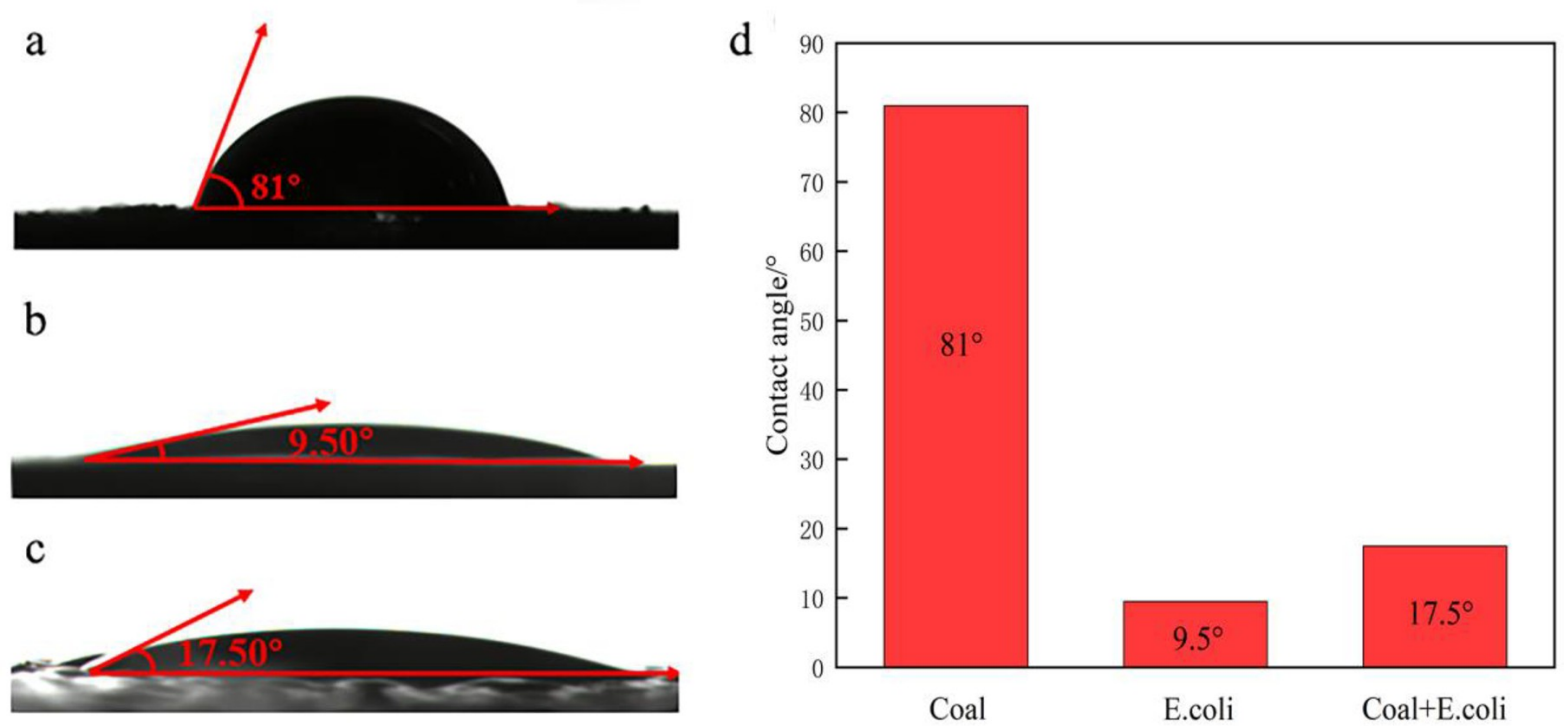
The contact angles of coal before and after interaction with *E*.*coli* (a) coal, (b) *E*.*coli*, (c) coal after interaction with *E*.*coli*, (d) The difference in contact angle before and after the interaction of coal with *E*. *coli*.

The contact angle can reflect the surface characteristics of *E*. *coli* and coal. Fig 4 shows the contact angles of coal before and after interaction with *E*.*coli* (5.0 × 10^9^ cells/ml) at pH 6. The surface of coal was hydrophobic prior to interaction with *E*.*coli*, and its contact angle was 81°. Due to the high hydrophobicity of the coal surface, the recovery of coal could reach 84.25% under the condition of flotation without collectors. The contact angle of *E*.*coli* was 9.5°, which implied that its surface had strong hydrophilicity. However, after interaction with *E*.*coli*, this coal gained hydrophilic characteristics, and its contact angle became17.9°. This can be attributed to the adsorbed bacterial cells onto the mineral surface, as they formed a biofilm that imparted their own surface properties to the mineral [23].

### 3.4 Zeta potential measurements

*E*.*coli* is a Gram-negative bacteria with cell wall, cell membrane, cytoplasm, ribosomes, plasmids, and nucleoids. The cell wall plays an important role in bacterial cells adsorbed onto the mineral surface. The anionic and cationic functional groups in the macromolecular molecules of cell wall gives the bacteria amphoteric characters which affect the bacterial adsorption [24].

**Fig 5:**
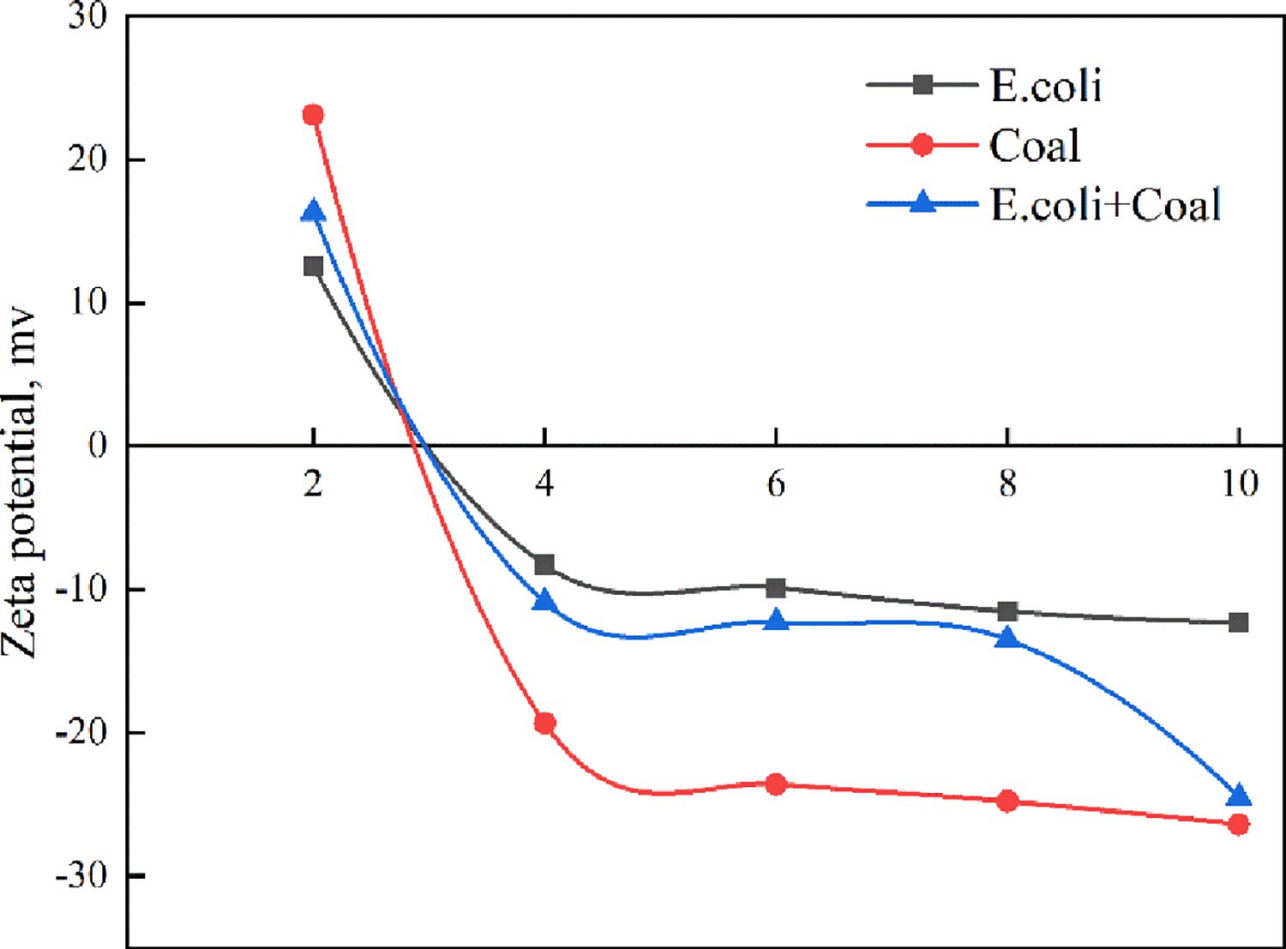
Zeta potential of coal before and after interaction with *E*.*coli* as a function of pH.

Zeta potential as a function of pH for coal before and after interaction with *E*.*coli* was tested, and the results are shown in Fig 5. When pH < 3, *E*.*coli* was positively charged. At PH 3, the anionic and cationic charges were in balance, which was the isoelectric point (IEP) of *E*. *coli*. When pH ranged from 3–10, *E*.*coli* was negatively charged, and the surface charge increased with increasing pH. At pH 10, the amount of charge reached a maximum value of 12 mV. According to the report, the negative charge of *E*.*coli* is primarily due to the presence of amino, carboxyl and phosphate groups in peptidoglycan and the IEP of *E*. *coli* has been shown to result from cell wall gluconic acids or other polysaccharide-associated carboxyl groups [25,26].

The IEP of coal was pH 2.8, when pH was 2.8, coal was positively charged, while at pH 2.8, coal was negatively charged, which agreed with previously reported data [27]. Under strong acidic, neutral and alkaline conditions, both *E*.*coli* and coal had the same electrical properties, so electrostatic interaction did not play a major role in the adsorption of *E*.*coli* on the coal surface. After interaction with *E*.*coli*, the IEP and zeta potential of the coal was close to that of *E*.*coli*, especially at pH 2–8. This indicated that the adsorption of *E*.*coli* onto the coal surface conferred the *E*.*coli* surface properties to the coal surface through the formation of a biofilm. At pH = 10, zeta potential of the coal after interaction with the *E*.*coli* was close to that of the coal. This may be due to the fact that the suitable pH range for the survival of *E*.*coli* is 4–8. At pH = 10, *E*. *coli* died and cannot be adsorbed on the surface of the coal. This showed that in a strong alkaline condition, *E*.*coli* was difficult to be adsorbed to the coal surface, but the extracellular polymeric substances (EPS) of bacteria could still change the hydrophobicity of the mineral and affect the flotation recovery [28]. The result of zeta potential could explain the effect of pH on the flotation recovery of coal with *E*.*coli*.

### 3.5 FTIR analysis

**Fig 6:**
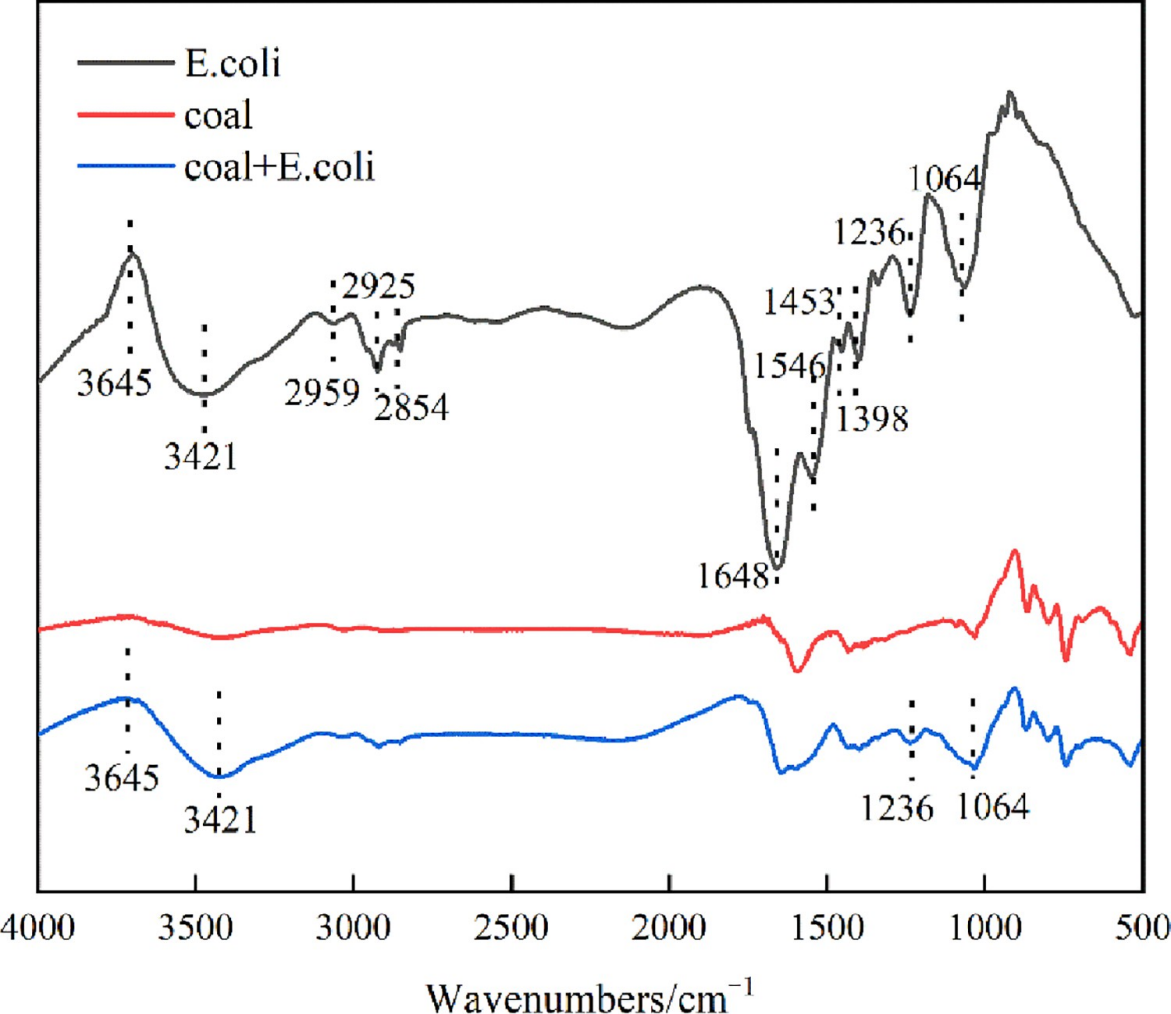
FTIR of coal and *E*.*coli* before and after their interaction.

Fig 6 shows the FTIR spectra of coal before and after interaction with *E*.*coli*. The intracellular and extracellular structures of microbial cells contain biological macromolecules such as polysaccharides, proteins and nucleic acids [22]. The information of the macromolecular structure in the cell can be characterized by infrared spectroscopy, and the adsorption of *E*.*coli* on the coal surface can be inferred [29]. In the spectrum of *E*.*coli*, the characteristic peaks at 3645cm^-1^ were due to the O-H stretching vibration of hydrogen bonding and -OH stretching vibration peak of sugar C-OH. The peaks at 3421cm^-1^ were due to the NH_2_ symmetrical stretching in protein molecules. The characteristic peaks at 2959, 2925 and 2854 cm^-1^ were due to CH_3_ asymmetric stretching fatty acids, CH_3_ symmetric stretching proteins, and CH_2_ symmetric stretching lipids respectively. The characteristic peaks at 1648, 1546 and 1453 cm^-1^ were due to amide I C = O stretching proteins, amide II N-H bending, C-N stretching proteins and CH_2_ bending lipids. The characteristic peaks at 1398 cm^-1^ were due to COO- symmetric stretching of amino acid side chains and fatty acids. The characteristic peaks at 1236 and 1064 cm^-1^ were due to C-O-C, C-O, C-O-P and P-O-P vibrations of polysaccharides [30].

The results of coal interaction with *E*.*coli* showed that the new peaks appeared near 3645, 3421, 1236, and 1064 cm^-1^, which were attributed to the characteristic peaks of *E*.*coli*. This indicated that when *E*.*coli* had an interaction with the coal, the groups in the polysaccharide and protein molecules in the cell wall of *E*.*coli* could form hydrogen bonds with the coal surface, so that *E*.*coli* could be adsorbed on the coal surface.

## 4. Conclusions

This study assessed the adverse effect of *E*.*coli* on coal flotation, the results showed that *E*.*coli* concentration affected the flotation recovery of coal. When *E*.*coli* concentration reached 5 × 10^9^ cells/ml, only 43% of the coal could be recovered. The pH value had little effect on the bioflotation of coal. Zeta potential results showed that under strong acidic, neutral and alkaline conditions, both *E*.*coli* and coal had the same electrical properties, so there was electrostatic repulsion between *E*.*coli* and coal surface which would hinder *E*.*coli* adsorption. But SEM, FTIR and Contact angle results showed that *E*.*coli* could be adsorbed to coal surface through hydrogen bonding, and then conferred its own surface characteristics to the coal surface by forming a biofilm. So it could be inferred that the hydrogen bonding force when *E*.*coli* adsorbed to the coal surface was greater than the electrostatic repulsion force. As the surface of coal was hydrophobic, and the surface of *E*.*coli* was hydrophilic, during the flotation process, *E*.*coli* adsorbed to the coal surface, which changed the hydrophobicity of the coal surface and affected the recovery of coal flotation. Many investigations have used known external bacteria as flotation reagents for bioflotation. In this study, the impact of internal bacteria of recirculation water on coal flotation was researched. Due to the negative effects of *E*.*coli* on coal flotation, it is necessary to remove *E*.*coli* from recirculation water before flotation.
